# Health Tract, No. 297

**Published:** 1867-08

**Authors:** 


### BRAIN WORK.

HARD study does not of itself shorten life, but does of itself tend to increase the longevity of man. When hard students die early, it will be found that in some way they had fallen into the habit of violating some of the laws of nature or began study with some inherited infirmity. The pursuit of Truth is pleasurable; it is exhilarating; it is exalting and promotes serenity. Of all men, natural philosophers average the longest lives. The great, the governing reason is, in addition to the above, that their attention is drawn away from the endulgence of animal appetites; their gratifications are not in that direction, hence they are neither gourmands, drunkards nor licentious. Sir Isaac Newton had often to be reminded that his dinner was waiting; the call to eat is oftan a most unwelcome one to literary men; they consider eating a secondary consideration; they literally eat to live, and the process of dining is often gone through with as a task.

Many hard students have become miserable dyspeptics and have died while yet in their prime, but the tormenting disease was brought on by over eating, by eating too fast, or by returning to their studies too soon after a hearty or hasty meal, thus drawing to the brain the nervous energy which ought to have been expended on the stomach in aiding it to prepare the food for nourishing the system, and not being so prepared it "lays heavy," feels like a load, or induces other discomforts which increase in intensity and duration until life becomes a burden and a failure. The Freuch Academy is perhaps the most learned body in the world and the ages of the younger members average from sixty to seventy. Most of the clever men of France have in this year of 1867, reached a great age. Of the members of the French Academy, M, Viennet is 89; M. de Segur 86; de Pougerville, 76; Lebrun, 82; Villemain, 76; Lamartine, 76; Flourene, 78; M. Guizot is 79 and M. Thiers 69; Berryer is 74; the Duke de Broglie, 82.

This list might be indefinitely extended as to all nations — Lord Brougham, Humbolt, John Wesley, and many others.

The circumstance most favorable to longevity among brain workers is the spending a considerable portion of early life in out-door activities, travel and the like, and then by a temperate and plain mode of living the brain will work advantageously until past four score years.

